# Sulforaphane Inhibited the Nociceptive Responses, Anxiety- and Depressive-Like Behaviors Associated With Neuropathic Pain and Improved the Anti-allodynic Effects of Morphine in Mice

**DOI:** 10.3389/fphar.2018.01332

**Published:** 2018-11-20

**Authors:** Pablo Ferreira-Chamorro, Alejandro Redondo, Gabriela Riego, Sergi Leánez, Olga Pol

**Affiliations:** ^1^Grup de Neurofarmacologia Molecular, Institut d’Investigació Biomèdica Sant Pau, Barcelona, Spain; ^2^Grup de Neurofarmacologia Molecular, Institut de Neurociències, Universitat Autònoma de Barcelona, Barcelona, Spain

**Keywords:** analgesia, anxiety, depression, Nrf2 transcription factor, opioids, oxidative stress, chronic pain

## Abstract

Chronic neuropathic pain is associated with anxiety- and depressive-like disorders. Its treatment remains a serious clinical problem due to the lack of efficacy of the available therapeutic modalities. We investigated if the activation of the transcription factor Nrf2 could modulate the nociceptive and emotional disorders associated with persistent neuropathic pain and potentiated the analgesic activity of morphine. The possible mechanisms implicated in these effects have been also evaluated. Therefore, in C57BL/6 mice with neuropathic pain induced by the chronic constriction of the sciatic nerve (CCI), we assessed the antinociceptive, anxiolytic, and anti-depressant effects of the repeated intraperitoneal administration of a Nrf2 inducer, sulforaphane (SFN), and the effects of this treatment on the local antinociceptive actions of morphine. The protein levels of Nrf2, heme oxygenase 1 (HO-1), NAD(P)H:quinone oxidoreductase-1 (NQO1), CD11b/c (a microglial activator marker), mitogen-activated protein kinases (MAPK) and μ opioid receptors (MOR) in the spinal cord, prefrontal cortex and hippocampus from mice, at 28 days after CCI, were also evaluated. Our results showed that the repeated administration of SFN besides inhibiting nociceptive responses induced by sciatic nerve injury also diminished the anxiety- and depressive-like behaviors associated with persistent neuropathic pain. Moreover, SFN treatment normalized oxidative stress by inducing Nrf2/HO-1 signaling, reduced microglial activation and JNK, ERK1/2, p-38 phosphorylation induced by sciatic nerve injury in the spinal cord and/or hippocampus and prefrontal cortex. Interestingly, treatment with SFN also potentiated the antiallodynic effects of morphine in sciatic nerve-injured mice by regularizing the down regulation of MOR in the spinal cord and/or hippocampus. This study suggested that treatment with SFN might be an interesting approach for the management of persistent neuropathic pain and comorbidities associated as well as to improve the analgesic actions of morphine.

## Introduction

Chronic pain, especially neuropathic, significantly alters human living conditions. That is, patients with neuropathic pain experience emotional disorders such as anxiety and depression that produce a significant deterioration in their quality of life (Maletic and Raison, 2009; Attal et al., 2011). Therapies to treat neuropathic pain and associated comorbidities are limited with modest efficacy and significant side effects. Therefore, the investigation of new strategies to effectively relieve neuropathic pain and the emotional disorders associated is indispensable.

It is well known that the transcription factor Nrf2 coordinates the redox state in the cell and its activation serves as a barrier against the reactive species of oxygen through the activation of antioxidant and detoxifying enzymes, such as heme oxygenase-1 (HO-1), NAD(P)H:quinone oxidoreductase1 (NQO1), glutathione peroxidase (GPx1) enzymes, etc…(de Vries et al., 2008). The transcription factor Nrf2 also modulates inflammatory responses of the organism by inhibiting the synthesis of various proinflammatory mediators, such as the inducible nitric oxide synthase (NOS2), cyclooxygenase-2, interleukin (IL)-1α, IL-6, and mitogen-activated protein kinase (MAPK), among others (Kim et al., 2009; Davidson et al., 2013; Kobayashi et al., 2016). Furthermore, diverse studies revealed that the stimulation of Nrf2 reduced acute and inflammatory pain as well as neuropathic pain accompanying to diabetes (Negi et al., 2011; Di et al., 2016; McDonnell et al., 2017; Redondo et al., 2017), but the potential inhibitory role played by this transcription factor on the allodynia and hyperalgesia caused by nerve injury-induced persistent neuropathic pain has not been evaluated.

Numerous studies have demonstrated the anxiolytic and anti-depressant effects induced by Nrf2 in several stress paradigms (Wu et al., 2016) and in different animal models of depression (Yao et al., 2016). Moreover, the fact that mice with the Nrf2 gene silenced by pharmacological or genetic tools exhibited greater anxiety- and depressive-like behaviors sustained the anxiolytic and anti-depressant properties induced by Nrf2 activation in different experimental conditions (Muramatsu et al., 2013; Khalifeh et al., 2015). Nonetheless, the effects of the repetitive treatment with sulforaphane (SFN), a Nrf2 inducer (de Figueiredo et al., 2015), in the anxiety- and depressive-like behaviors associated with chronic pain have not yet studied. Our objective was to evaluate if the activation of Nrf2 besides reducing nociception might also inhibited the anxiety- and depressive-like behaviors associated with persistent neuropathic pain and normalized oxidative stress and microglial activation induced by sciatic nerve injury.

Sulforaphane is an isothiocyanate bioactive metabolite derived from glucoraphanin which is abundantly found in cruciferous vegetables (Dinkova-Kostova et al., 2017; Fahey et al., 2017). Several studies have demonstrated the potent anti-inflammatory, antioxidant, anti-cancer, antibiotic, as well as the protective effects of SFN for cognitive and memory impairments (Zhang et al., 1994; Guerrero-Beltran et al., 2012; Lee et al., 2014; Catanzaro et al., 2017; Pu et al., 2018). SFN has been also proven in humans demonstrating that this treatment improves the glucose levels in patients with type 2 diabetes (Axelsson et al., 2017), has beneficial clinical effects against autism disorders (Singh et al., 2014) and gastrointestinal diseases (Yanaka, 2018). Recent studies also showed the analgesic properties of SFN in several animal pain models and its capacity to potentiate the antinociceptive effects of opioids in animals with inflammatory pain or diabetic neuropathy (McDonnell et al., 2017; Redondo et al., 2017). Taking account the lower efficacy of opioids, particularly μ-opioid receptor (MOR) agonists in the management of neuropathic pain (Hervera et al., 2013; Zychowska et al., 2013; Popiolek-Barczyk and Mika, 2016), the investigation of new tools for increasing their analgesic efficacy is a priority in the current pain research. In this study we evaluated the possible potentiation of the analgesic actions of morphine triggered by SFN in animals with persistent neuropathic pain.

Then, in a neuropathic pain model induced by the chronic constriction of sciatic nerve (CCI) in mice, at 28 days after surgery, our aims were to assess the effects of treatment with SFN on: (1) the allodynia and hyperalgesia induced by CCI; (2) the anxiety- and depressive-like behaviors associated with persistent neuropathic pain; (3) the local antinociceptive effects of morphine during neuropathic pain and (4) the expression of Nrf2/HO-1/NQO1 signaling pathway, microglial activation, MAPK phosphorylation and MOR protein levels in the spinal cord, prefrontal cortex and hippocampus from animals with persistent neuropathic pain.

## Materials and Methods

### Animals

The experiments were carried out with male C57BL/6J mice purchased from Envigo Laboratories (Barcelona, Spain). Mice weighing 21–25 g were housed under standard 12/12-h light/dark conditions in a room with a controlled temperature of 22°C and relative humidity of 66%. The animals had free access to food and drink and were used after 6 days of acclimatization to the environmental conditions mentioned. All experiments were conducted between 9:00 a.m. and 5:00 p.m., and executed in accordance with the National Institute of Health Guide for the Care and Use of Laboratory Animals and approved by the local Committee of Animal Use and Care of the Autonomous University of Barcelona. All efforts were made to diminish the suffering and amount of animals used in this study.

### Induction of Neuropathic Pain

Neuropathic pain was induced by CCI (Hervera et al., 2012). Briefly, sciatic nerve ligation was performed under isoflurane anesthesia (3% induction, 2% maintenance). The biceps femoris and the gluteus superficialis were separated by blunt dissection, and the right sciatic nerve was exposed. The injury was produced by tying three ligatures around the sciatic nerve as described by Bennett and Xie (1988). The ligatures (4/0 silk) were tied loosely around the nerve with 1 mm spacing, until they elicited a brief twitch in the respective hindlimb, which prevented overtightening of the ligations, taking care to preserve epineural circulation. In these experiments Sham-operated mice, whose surgery was exactly the same as described above without sciatic nerve ligament, were used as control animals.

### Experimental Protocol

In a first set of experiments, we investigated the mechanical antiallodynic, thermal antihyperalgesic and thermal antiallodynic effects of the intraperitoneal daily administration of 10 mg/kg SFN in sciatic nerve-injured or Sham-operated mice from days 14 to 28 after surgery (*n* = 6 animals per group). The evaluation of the antinociceptive effect was carried out on days 14, 18, 21, 25, and 28 post-surgery, at 3 h after SFN or vehicle injection.

In other group of animals, we evaluated the effects of treatment with SFN, also administered at 10 mg/kg during 15 consecutive days (from days 14 to 28 post-surgery), on the anxiety- and depressive-like responses associated with persistent neuropathic pain, 28 days after surgery, using the EPM test and TST, respectively (*n* = 8 animals per group).

In other experiments, the evaluation of the effects produced by the intraperitoneal injection of 10 mg/kg of SFN combined with 50 μg of morphine, subplantarly administered, on the allodynia and hyperalgesia induced by CCI was carried out at 3 h after SFN administration (*n* = 6 animals per group). The doses of SFN and morphine were selected in accordance to other studies (Redondo et al., 2017; Wang and Wang, 2017).

Finally, at day 15 of treatment with SFN or vehicle (dimethylsulfoxide 1% in 0.9% saline solution), sciatic nerve-injured and Sham-operated mice were euthanized by cervical dislocation and the Nrf2, HO-1, NQO1, CD11b/c, MAPK, and MOR levels in the ipsilateral site of the spinal cord, prefrontal cortex and hippocampus were evaluated. In these experiments, Sham-operated mice treated with vehicle were used as controls (*n* = 4 samples per group).

The number of animals per group was determined from a pilot study taking into account a value of α = 0.05 and β = 0.20 (power analysis of 0.80). In this work we used 60 animals in total.

### Nociceptive Behavioral Tests

#### Mechanical Allodynia

Mechanical allodynia was quantified by measuring the hind paw withdrawal response to von Frey filament stimulation. Animals were placed in methacrylate cylinders (20 cm high × 9 cm diameter) with a wire grid bottom through which the von Frey filaments (North Coast Medical, Inc., San Jose, CA, United States), with a bending force in the range of 0.008–3.5 g, were applied by using a modified version of the up–down paradigm, described by Chaplan et al. (1994). The filament of 0.4 g was used first and the 3.0 g filament was used as a cut-off and the strength of the next filament was reduced or increased according to the response. The threshold of response was calculated from the sequence of filament strength used during the up-down procedure by using an Excel program (Microsoft Iberia SRL, Barcelona, Spain) that includes curve fitting of the data. Both ipsilateral and contralateral hind paws were tested. Animals were allowed to habituate for 1 h prior to testing to allow appropriate behavioral immobility.

#### Thermal Hyperalgesia

Thermal hyperalgesia was evaluated as proposed by Hargreaves et al. (1988). Latency of paw withdrawal in response to radiant heat was measured using the plantar test apparatus (Ugo Basile, Varese, Italy). In summary, mice were placed in methyl acrylate cylinders (20 cm high × 9 cm diameter) positioned on a glass surface. The heat source was positioned under the plantar surface of the hind paw and activated with a light beam intensity. A cut-off time of 12 s was used to avoid tissue damage in the absence of response. Mean paw withdrawal latencies (from both hind paws) were determined from the average of three separate trials, taken at 5 min intervals to avoid thermal sensitization and behavioral alterations. Mice were habituated to the environment for 1 h prior to the experiment so that the animals were quiet at the time of testing.

#### Thermal Allodynia

Thermal allodynia to cold stimulus was evaluated using the hot/cold plate analgesia meter (Ugo Basile) previously described by Bennett and Xie (1988). The number of elevations of each hind paw from mice exposed to the cold plate (4 ± 0.5°C) was recorded for 5 min.

### Anxiety-Like Behavior

The anxiety-like behavior was measured by using the elevated plus maze (EPM) test such as described by Walf and Frye (2007). An apparatus with 4 arms of 5 cm wide and 35 cm long, two of which are open and two closed with walls of 15 cm high. The distance of the EPM to the ground is 45 cm. The animal was placed in the central square of maze facing one of the open arms and its behavior was recorded by a digital camera for 5 min. The number of entries in the open and closed arms, as well as the percentage of time spent in the open arms was calculated for each animal.

### Depressive-Like Behavior

The evaluation of the depressive-like behavior was performed by using the tail suspension test (TST), in which the total duration of immobility of the animals was quantified according to the method described by Steru et al. (1985) with some modifications. Briefly, mice were individually suspended by the tail from a horizontal wooden bar (35 cm above the floor) using an adhesive tape (1 cm from the tip of the tail). The immobility time in seconds was recorded over a total period of 6 min.

All the behavioral experiments were performed by an experimenter blinded to the treatment applied.

### Western Blot Analysis

Sham-operated and sciatic nerve-injured mice were killed at 28 days after surgery by cervical dislocation and tissues from the ipsilateral side lumbar section of the spinal cord, prefrontal cortex and hippocampus were extracted immediately after killing, frozen in liquid nitrogen and stored at -80°C. Samples from two to three animals were collected in an experimental sample to obtain enough protein levels for doing western blot analysis of Nrf2, HO-1, NQO1, CD11b/c, MAPK (JNK, ERK1/2, and P38) and MOR. Tissues were homogenized in ice-cold lysis buffer (50 mM Tris⋅Base, 150 nM NaCl, 1% NP-40, 2 mM EDTA, 1 mM phenylmethylsulfonyl fluoride, 0.5 Triton X-100, 0.1% sodium dodecyl sulfate, 1 mM Na_3_VO_4_, 25 mM NaF, 0.5% protease inhibitor cocktail, and 1% phosphatase inhibitor cocktail). All reagents were purchased from Sigma (St. Louis, MO) excluding NP-40 which was acquired from Calbiochem (Darmstadt, Germany). The crude homogenate was solubilized for 1 h at 4° C, sonicated for 10 s and centrifuged at 4°C for 15 min at 700 g.

Then, 60 μg of total proteins were mixed with 4 × laemmli loading buffer and loaded onto 4% stacking/10% separation sodium dodecyl sulfate polyacrylamide gels. Proteins were electrophoretically transferred onto PVDF membrane for 120 min, blocked with PBS or TBST + 5% non-fat dry milk or BSA and successively incubated overnight at 4°C with rabbit anti Nrf2 (1:160, Abcam, Cambridge, United Kingdom), HO-1 (1:300, Abcam, Cambridge, United Kingdom), NQO1 (1:333, Sigma, St. Louis, MO, United States), CD11b/c (1:200, Novus Biologicals, Littleton, CO, United States), phospho JNK, total JNK, phospho ERK1/2, total ERK1/2, phospho P38 and total P38 (1:250, Cell Signaling Technology, Danvers, MA, United States), MOR (1:333, Merck, Billerica, MA, United States) or GAPDH antibody (1:5000, Merck, Billerica, MA, United States) which was used as a loading control. Proteins were detected by a horseradish peroxidase-conjugated anti-rabbit secondary antibody (GE Healthcare, Little Chalfont, Buckinghamshire, United Kingdom) and visualized with chemiluminescent reagents (ECL kit; GE Healthcare) and by exposure to hyperfilm (GE Healthcare). The intensity of blots was quantified by densitometry.

### Drugs

Sulforaphane was acquired from Merck Chemicals and Life Science S.A.U. (Madrid, Spain), dissolved in dimethylsulfoxide (1% in 0.9% saline solution) and intraperitoneally administered at 10 mg/kg, in a final volume of 10 ml/kg, 3 h before testing. Morphine hydrochloride was purchased from Alcaiber S.A. (Madrid, Spain), dissolved in 0.9% saline solution and administered via subplantar, in a final volume of 30 μl, at 30 min before conducting the behavioral tests. All drugs were prepared daily prior to its administration. For each group treated with a drug, the respective control group received the same volume of the corresponding vehicle.

### Statistical Analysis

All data were expressed as mean ± SEM. Statistical analysis was carried out using the SPSS program (version 17 for Windows, IBM, Madrid, Spain). The effects of chronic treatment with SFN on the mechanical allodynia, thermal hyperalgesia and thermal allodynia induced by CCI were evaluated using the three way analysis of variance (ANOVA) repeated measures followed by a one way ANOVA and the Student–Newman–Keuls test, when appropriate. The antinociceptive effects produced by the combination of SFN plus morphine were assessed by using a one way ANOVA followed by the Student–Newman–Keuls test.

The effects of chronic administration with SFN on the anxiety-like behavior identified by the number of open arm entries, the percentage of time of stay in them and the number of closed arm entries obtained in EPM as well as on the depressive-like behavior defined by the immobility time in TST, in Sham and animals with neuropathic pain, were evaluated by using a two way ANOVA followed by a one way ANOVA and the Student–Newman–Keuls test.

Antinociception in the von Frey filaments and plantar tests is expressed as the percentage of maximal possible effect, where the test latencies predrug (baseline) and postdrug administration are compared and calculated in accordance with the following equation:

(1) $$\begin{array}{c} Maximal possible effect(\%)=[(drug-baseline)/(\mathrm{cut}-\mathrm{off}-\mathrm{baseline})]\times 100 \end{array}$$

In the cold plate, antinociception is expressed according to the following equation:

(2) $$\begin{array}{c} \mathrm{Inhibition}(\%)=[(\mathrm{number of paw elevations at baseline}-\mathrm{number of paw elevations after drug})/\mathrm{number of paw elevations at baseline})]\times 100. \end{array}$$

Changes in the protein levels were analyzed using a one way ANOVA followed by the Student–Newman–Keuls. A value of *P* < 0.05 was considered significant.

## Results

### Effects of SFN on the Mechanical Allodynia, Thermal Hyperalgesia and Thermal Allodynia Induced by Sciatic Nerve Injury in Mice

**FIGURE 1:**
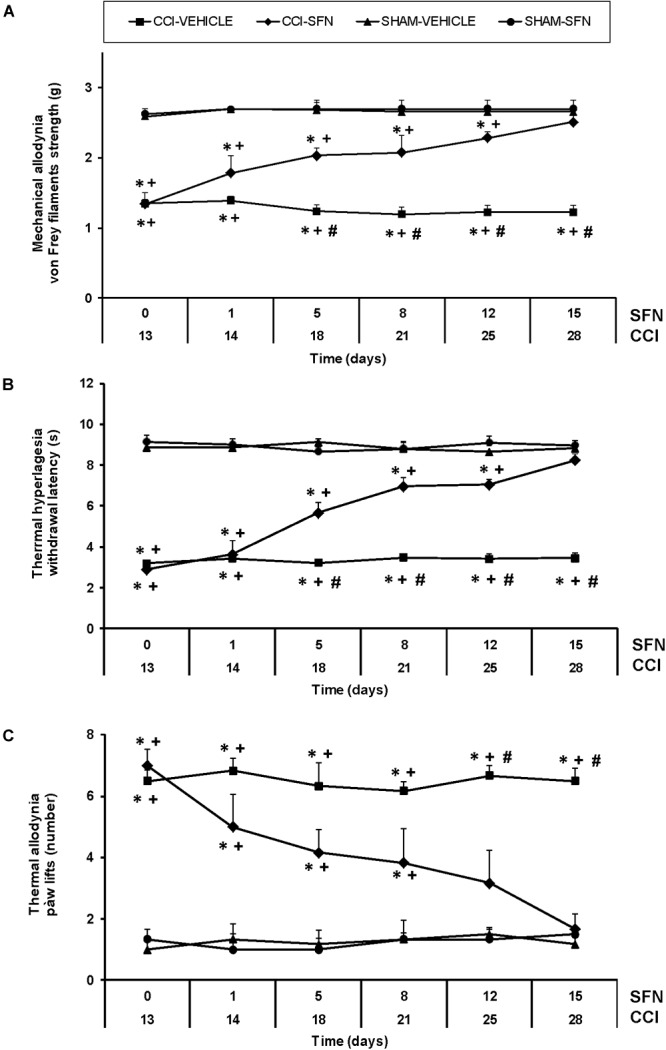
The antinociceptive effects of SFN during neuropathic pain. Mechanical antiallodynic **(A)**, thermal antihyperalgesic **(B)**, and thermal antiallodynic **(C)** effects produced by the repeated administration of 10 mg/kg SFN or vehicle from days 14 to 28 after sciatic nerve-injury (CCI) or Sham-operation (SHAM). In all panels, for each day and treatment evaluated, ^∗^ indicates significant differences vs. Sham-operated animals treated with vehicle, + vs. Sham-operated animals treated with SFN and # vs. sciatic nerve-injured animals treated with SFN (*P* < 0.05, one way ANOVA followed by Student–Newman–Keuls test). Results are shown as mean values ± SEM; *n* = 6 animals per experimental group.

For mechanical allodynia, the three way ANOVA repeated measures indicated significant effects of surgery, treatment and time (*P* < 0.022), an interaction of treatment with time (*P* < 0.007), surgery with treatment (*P* < 0.001) and among surgery, treatment and time (*P* < 0.006). Indeed, nerve injury reduced the threshold of paw withdrawal to a mechanical stimulus from days 13 to 28 after surgery as compared to Sham-operated mice treated with vehicle (*P* < 0.001, one way ANOVA) (Figure 1A). This mechanical allodynia was progressively reduced from 1 to 12 days of SFN administration (*P* < 0.001; one way ANOVA vs. CCI-vehicle treated mice) and completely inhibited at 15 days of treatment. In contrast, SFN did not produce any effect in Sham-operated mice for the total duration of the experiment.

Sciatic nerve injury also significantly decreased threshold for evoking paw withdrawal to a thermal stimulus from days 13 to 28 after surgery (*P* < 0.001; one way ANOVA vs. Sham-operated mice) (Figure 1B). Three way ANOVA repeated measures showed significant actions of surgery, treatment and time (*P* < 0.001) and interactions among treatment and time (*P* < 0.001), surgery and treatment (*P* < 0.001), surgery and time (*P* < 0.001) and between the three factors (*P* < 0.001). Thermal hyperalgesia induced by CCI was progressively reduced from 1 to 12 days of SFN administration (*P* < 0.001; one way ANOVA vs. CCI-vehicle treated mice) and completely reversed after 15 days of treatment (Figure 1B). In Sham-operated mice, SFN did not cause any effect throughout the experiment.

Regarding thermal allodynia, three way ANOVA repeated measures also demonstrated relevant effects of surgery and time (*P* < 0.011) and interactions among treatment and time (*P* < 0.05) as well as between surgery and treatment (*P* < 0.016) (Figure 1C). That is, increased paw lifts number induced by cold thermal stimulation was observed in CCI-mice from days 13 to 28 after surgery (*P* < 0.001; one way ANOVA vs. Sham-operated mice). This thermal allodynia was progressively reduced from 1 to 12 days of SFN treatment (*P* < 0.001; one way ANOVA vs. CCI-vehicle treated mice), and totally reversed at 15 days of treatment. SFN did not produce any effect in Sham-operated mice during the course of the experiment. In all tests, the administration of SFN did not affect the contralateral paw of sciatic nerve-injured or Sham-operated mice (data not shown).

### Effects of Chronic Administration With SFN on the Anxiety- and Depressive-Liked Behaviors Associated With Persistent Neuropathic Pain in Mice

**FIGURE 2:**
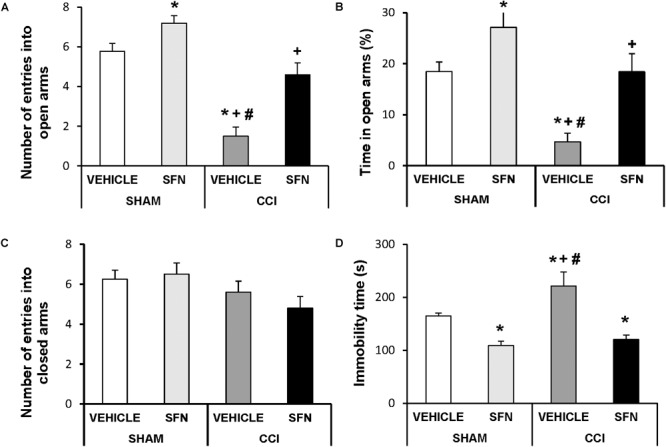
The anxiolytic and anti-depressant effects induced by SFN in animals with neuropathic pain. Effects of the repetitive administration of 10 mg/kg SFN or vehicle from days 14 to 28 after sciatic nerve injury (CCI) or Sham-operation (SHAM) on the anxiety- (elevated plus maze, **A–C**) and depressive-liked (tail suspension test, **D**) behaviors associated with neuropathic pain. In all panels, ^∗^ indicates significant differences vs. Sham-operated animals treated with vehicle, + vs. Sham-operated animals treated with SFN and # vs. sciatic nerve-injured animals treated with SFN (*P* < 0.05, one way ANOVA followed by Student–Newman–Keuls test). Results are shown as mean values ± SEM; *n* = 8 animals per experimental group.

**FIGURE 3:**
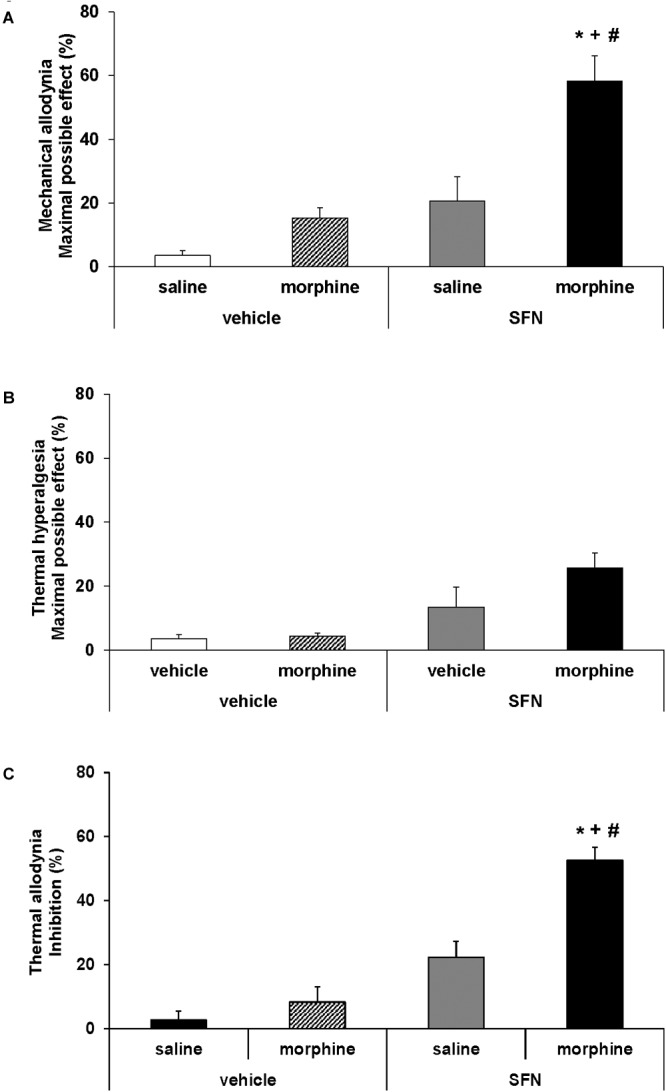
Effects of SFN on the local antinociceptive effects of morphine during neuropathic pain. The mechanical antiallodynic **(A)**, thermal antihyperalgesic **(B)**, and thermal antiallodynic **(C)** effects produced by the acute administration of 10 mg/kg SFN or vehicle combined with a subanalgesic dose (50 μg) of morphine or saline administered on the ipsilateral paw of sciatic nerve-injured mice at 28 days after surgery were shown. In all panels,^∗^ indicates significant differences vs. vehicle plus saline treated mice, + vs. vehicle plus morphine treated mice and # vs. SFN plus saline treated mice (*P* < 0.05, one way ANOVA followed by Student–Newman–Keuls test). Results are shown as mean values ± SEM; *n* = 6 animals per experimental group.

Anxiety-like behavior was observed in sciatic nerve-injured animals at day 28 post-CCI. The two way ANOVA revealed a significant effect of the surgery ant treatment on the number of entries and on the percentage of time of animals spent in open arms (*P* < 0.001). That is, the EPM test showed a significant decrease in the number of entries into open arms (Figure 2A, *P* < 0.05, ANOVA) and a reduced percentage of the time spent in these arms in sciatic nerve-injured mice treated with vehicle (Figure 2B; one way ANOVA; as compared to Sham-operated mice treated with vehicle). No differences in the number of entries to the closed arms were observed between sciatic nerve-injured and Sham-operated mice treated with SFN or vehicle (Figure 2C). Chronic treatment with SFN inhibited this anxiety-like behavior by normalizing the decreased number of entries into open arms (Figure 2A; *P* < 0.001; ANOVA) and the reduced time spent in open arms (Figure 2B; *P* < 0.01, ANOVA) observed in CCI vehicle treated mice, without altering the number of entries to the closed arms (Figure 3C). In Sham-operated mice, SFN also had an anxiolytic effect as demonstrated by an increase in the number of entries (Figure 2A) and the time spent in open arms as compared to Sham-operated vehicle treated mice (Figure 2B; *P* < 0.05, ANOVA).

Regarding the TST test, significant effects of surgery (*P* < 0.033) and treatment (*P* < 0.001) have been also demonstrated by the two way ANOVA. Indeed, sciatic nerve injury induced a depressive-like behavior in the TST, proved by a significant increase in the immobility time (*P* < 0.05; one way ANOVA, as compared to Sham-operated mice treated with vehicle) (Figure 2D), which was significantly reduced by the administration of SFN. This treatment also decreased the immobility time in Sham-operated mice confirming its anti-depressant effects (*P* < 0.05; one way ANOVA, as compared to Sham-operated mice treated with vehicle).

### Effects of SFN Combined With Morphine on the Nociception Induced by Sciatic Nerve Injury in Mice

The co-administration of SFN with morphine in animals with CCI-induced neuropathic pain resulted in a significant increase in the mechanical (Figure 3A; one way ANOVA vs. mice treated with vehicle, morphine or SFN alone) and thermal antiallodynic effects of morphine (Figure 3C; *P* < 0.008; one way ANOVA vs. mice treated with vehicle, morphine or SFN alone). In contrast, no significant changes in the antihyperalgesic effects of morphine were observed in SFN treated mice (Figure 3B).

### Effects of SFN Treatment on the Expression of Nrf2, HO-1, NQO1, CD11b/c, MOR, MAPK in the Spinal Cord, Prefrontal Cortex and Hippocampus of Animals With Neuropathic Pain

**FIGURE 4:**
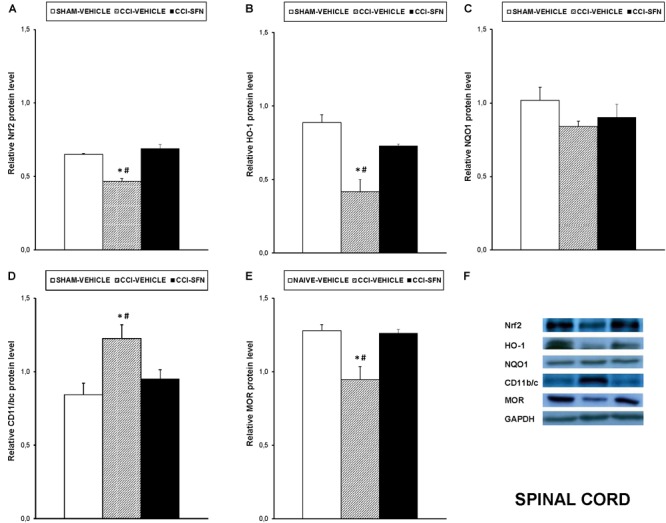
Effects of SFN on the expression of Nrf2, HO-1, NQO1, CD11b/c, and MOR in the spinal cord from animals with neuropathic pain. Effects of repetitive treatment with 10 mg/kg SFN or vehicle from days 14 to 28 after sciatic nerve injury (CCI) on Nrf2 **(A)**, HO-1 **(B)**, NQO1 **(C)**, CD11b/c **(D)**, and MOR **(E)** protein levels in the ipsilateral site of the spinal cord from CCI-induced neuropathic pain in mice are represented. The protein levels from Sham-operated (SHAM) mice treated with vehicle has been also represented as controls. Examples of western blots for Nrf2 (75 kDa), HO-1 (32 kDa), NQO1 (28 kDa), CD11b/c (160 kDa), and MOR (50 kDa) proteins in which GAPDH (36 kDa) was used as a loading control are also shown **(F)**. In all panels, ^∗^ indicates significant differences vs. Sham vehicle treated mice and # indicates significant differences vs. CCI plus SFN treated mice (*P* < 0.05, one-way ANOVA followed by Student–Newman–Keuls test). Data are expressed as mean values ± SEM; *n* = 4 samples per group.

**FIGURE 5:**
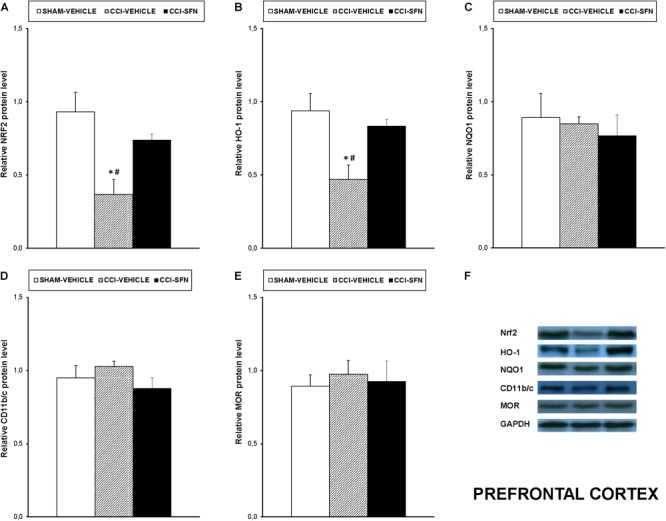
Effects of SFN on the expression of Nrf2, HO-1, NQO1, CD11b/c, and MOR in the prefrontal cortex from animals with neuropathic pain. Effects of repetitive treatment with 10 mg/kg SFN or vehicle from days 14 to 28 after sciatic nerve injury (CCI) on Nrf2 **(A)**, HO-1 **(B)**, NQO1 **(C)**, CD11b/c **(D)**, and MOR **(E)** protein expression in the prefrontal cortex from CCI-induced neuropathic pain in mice are represented. The protein levels from Sham-operated (SHAM) mice treated with vehicle has been also represented as controls. Examples of western blots for Nrf2 (75 kDa), HO-1 (32 kDa), NQO1 (28 kDa), CD11b/c (160 kDa), and MOR (50 kDa) proteins in which GAPDH (36 kDa) was used as a loading control are also shown **(F)**. In all panels, ^∗^ indicates significant differences vs. Sham vehicle treated mice and # indicates significant differences vs. CCI plus SFN treated mice (*P* < 0.05, one way ANOVA followed by Student–Newman–Keuls test). Data are expressed as mean values ± SEM; *n* = 4 samples per group.

**FIGURE 6:**
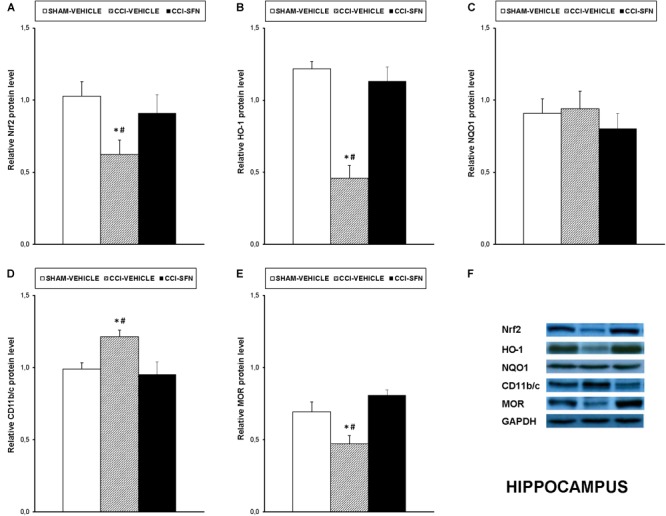
Effects of SFN on the expression of Nrf2, HO-1, NQO1, CD11b/c, and MOR in the hippocampus from animals with neuropathic pain. Effects of repetitive treatment with 10 mg/kg SFN or vehicle from days 14 to 28 after sciatic nerve injury (CCI) on Nrf2 **(A)**, HO-1 **(B)**, NQO1 **(C)**, CD11b/c **(D)**, and MOR **(E)** protein expression in the hippocampus from CCI-induced neuropathic pain in mice are represented. The protein levels from Sham-operated (SHAM) mice treated with vehicle has been also represented as controls. Examples of western blots for Nrf2 (75 kDa), HO-1 (32 kDa), NQO1 (28 kDa), CD11b/c (160 kDa), and MOR (50 kDa) proteins in which GAPDH (36 kDa) was used as a loading control are also shown **(F)**. In all panels, ^∗^ indicates significant differences vs. Sham vehicle treated mice and # indicates significant differences vs. CCI plus SFN treated mice (*P* < 0.05, one way ANOVA followed by Student–Newman–Keuls test). Data are expressed as mean values ± SEM; *n* = 4 samples per group.

Our results showed that treatment with SFN normalized the decreased expression of Nrf2 induced by sciatic nerve-injury in the spinal cord (*P* < 0.001; one way ANOVA, Figure 4A), prefrontal cortex (*P* < 0.013; one way ANOVA, Figure 5A) and hippocampus (*P* < 0.030; one way ANOVA, Figure 6A) as compared to their respective Sham-operated vehicle treated mice. SFN treatment was also able to normalize the decreased expression of HO-1 induced by CCI in the spinal cord (*P* < 0.008; one way ANOVA, Figure 4B), prefrontal cortex (*P* < 0.032; one way ANOVA, Figure 5B) and hippocampus (*P* < 0.039; one way ANOVA, Figure 6B). In contrast, the NQO1 levels were not significantly altered in the spinal cord (Figure 4C), prefrontal cortex (Figure 5C) or hippocampus (Figure 6C) from animals with neuropathic pain.

Nevertheless, SFN reduced the increased expression of CD11b/c induced by CCI in the spinal cord (*P* < 0.028; one way ANOVA, Figure 4D) and hippocampus (*P* < 0.021; one way ANOVA, Figure 6D). Finally, the decreased expression of MOR induced by nerve injury in the spinal cord (*P* < 0.012, one way ANOVA vs. Sham-operated vehicle treated mice; Figure 4E) and hippocampus (*P* < 0.018, one way ANOVA vs. Sham-operated vehicle treated mice; Figure 6E) was also normalized by SFN treatment. Non-changes in the expression of CD11b/c (Figure 5D) or MOR (Figure 5E) were observed in the prefrontal cortex of sciatic nerve-injured mice treated with vehicle or SFN. Examples of western blots of these proteins in the spinal cord (Figure 4F), prefrontal cortex (Figure 5F), and hippocampus (Figure 6F) were shown.

**FIGURE 7:**
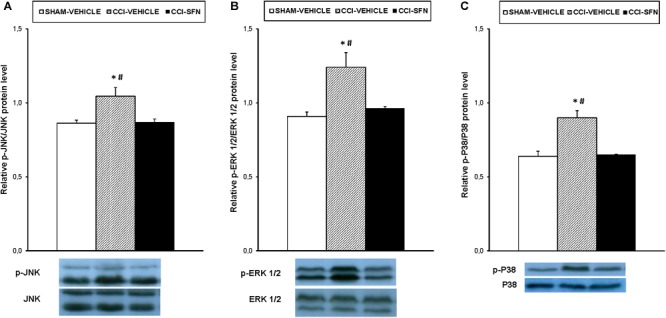
Effect of SFN on the expression of MAPK in the spinal cord from animals with neuropathic pain. Effects of repetitive treatment with 10 mg/kg SFN or vehicle from days 14 to 28 after sciatic nerve injury (CCI) on the protein levels of p-JNK **(A)**, p-ERK ½ **(B)**, and p-P38 **(C)** in the spinal cord are represented. The protein levels from Sham-operated (SHAM) mice treated with vehicle has been also represented as controls. In all panels, ^∗^ indicates significant differences vs. Sham-operated vehicle treated mice and # indicates significant differences vs. CCI-SFN treated mice (*P* < 0.05, one way ANOVA followed by Student–Newman–Keuls test). Each column represents the mean and vertical bars indicate the standard error of the mean (SEM); *n* = 4 samples per group. Representative examples of western blots for p-JNK/total JNK protein (46–54 kDa), p-ERK ½/total ERK ½ (42–44 kDa) and p-P38/total P38 (38 kDa) are also shown.

The effects of the repeated treatment with SFN on the expression of MAPK in the spinal cord from sciatic nerve-injured mice have been also evaluated. Results showed that the augmented protein levels of p-JNK (Figure 7A), p-ERK ½ (Figure 7B, and p-P38 (Figure 7C) induced by sciatic nerve injury (*P* < 0.012; one way ANOVA vs. Sham-operated vehicle treated mice) were all normalized by SFN treatment.

## Discussion

This study demonstrated that SFN besides inhibiting nociceptive responses induced by sciatic nerve injury also diminished the anxiety- and depressive-like behaviors associated with persistent neuropathic pain, at 28 days after surgery. Moreover, the normalization of oxidative stress, microglial activation and/or MAPK phosphorylation produced by SFN in the spinal cord, hippocampus and/or prefrontal cortex of sciatic nerve-injured mice might explain the analgesic, anxiolytic, and anti-depressant actions of this treatment. In addition, treatment with SFN also potentiated the antiallodynic effects of morphine by normalizing the down regulation of MOR in the spinal cord and/or hippocampus from sciatic nerve-injured mice.

Our results showed that the mechanical and thermal allodynia as well as the thermal hyperalgesia caused by sciatic nerve injury, at 28 days after induction, were inhibited by the repetitive administration of SFN from day 14 to 28 after sciatic nerve injury in mice. These results are supported by the antinociceptive effects produced by SFN in the early stages of neuropathic pain, from days 7 to 14 after surgery (Wang and Wang, 2017) as well as during inflammatory pain or diabetic neuropathy (McDonnell et al., 2017; Redondo et al., 2017), and additionally demonstrated the analgesic properties of this Nrf2 inductor in advance stages of neuropathic pain.

It is well known that Nrf2 transcription factor is a regulator of two important cytoprotective pathways: anti-oxidative and anti-inflammatory in the peripheral and central nervous system (Kobayashi et al., 2016). In order to study the possible mechanisms involved in the antinociceptive actions induced by SFN in animals with persistent neuropathic pain, we evaluated the effects of this treatment on the expression of Nrf2, the antioxidant enzymes (HO-1 and NQO1) as well as on the protein levels of CD11b/c (a microglial marker) in the spinal cord from animals with persistent neuropathic pain. In agreement with Riego et al. (2018), peripheral nerve injury provoked prevailing oxidative stress conditions in the spinal cord as demonstrated by the decreased expression of Nrf2 and HO-1 in this tissue. Interestingly, treatment with SFN reduced oxidative stress by normalizing the decreased expression of Nrf2 and HO-1. In accordance to these data, Riego et al. (2018) also demonstrated that treatment with an HO-1 inducer, cobalt protoporphyrin IX (CoPP), was able to compensate for the reduced synthesis of HO-1 induced by neuropathic pain in this tissue. Taking account that HO-1 is downstream of Nrf2 and its induction in the spinal cord exerts potent antinociceptive effects during chronic pain (Liu et al., 2016), it is suitable to propose that the antinociceptive effects induced by SFN during persistent neuropathic pain were principally associated with the activation of the Nrf2/HO-1 signaling pathway.

On the contrary, the protein levels of NQO1 were not altered by either nerve injury or SFN treatment in animals with neuropathic pain. This is in contrast to that happens during inflammatory pain or diabetic neuropathy, where SFN increased the expression of NQO1 or re-established its down-regulation in the spinal cord or sciatic nerve, respectively (McDonnell et al., 2017; Redondo et al., 2017). Thus, suggesting that this enzyme is not implicated in the antinociceptive effects of SFN in sciatic nerve injury induced neuropathic pain.

It is well known that microglial activation promotes the consolidation and progression of neuropathic pain state by the up-regulation of several inflammatory mediators, such as the inducible nitric oxide synthase (NOS2), COX-2, interleukins, etc. (Popiolek-Barczyk and Mika, 2016). In this study, we demonstrated that spinal microglial activation induced by nerve injury, at 28 days after surgery, was inhibited by SFN indicating that, in addition to the antioxidative effects performed by this treatment, it also exerted anti-inflammatory effects in this tissue contributing all of them to the inhibition of neuropathic pain. In agreement with our results, other previous studies also demonstrated anti-inflammatory effects of SFN in chronic pain that were produced by inhibiting of NOS2 and COX-2 expression in sciatic nerves from nerve-injured mice or diabetic animals (Negi et al., 2011; Wang and Wang, 2017).

It is well recognized that MAPK, such as JNK, ERK ½ and p38, are involved in pain sensitization after nerve injury. Moreover, while their phosphorylation participated in the maintenance of pain hypersensitivity, its inhibition attenuated chronic pain (Ji et al., 2009; Edelmayer et al., 2014). The fact that SFN inhibited the phosphorylation of JNK, ERK ½ and p38 in the spinal cord of sciatic nerve-injured mice showed that the inhibition of MAPK might be also involved in the anti-allodynic and anti-hyperalgesic effects of this treatment during persistent neuropathic pain. In according to these data, this signaling pathway was also involved in the analgesic effects of SFN during inflammatory pain (Redondo et al., 2017). In summary, the modulation of persistent sciatic nerve injury-induced neuropathic pain with the repeated administration of SFN might be explained by the activation of Nrf2/HO-1 signaling and the inhibition of microglial activation and MAPK phosphorylation induced by this treatment in the spinal cord.

In accordance with other preclinical pain models, our data verified that neuropathic pain induced by CCI, at 28 days after surgery, was associated with anxiety- and depressive-like behaviors (Jesse et al., 2010; Zhao et al., 2014; Alba-Delgado et al., 2016). That is, while anxiety-like response was demonstrated by the reduced number of entries and time spent into open arms in the EPM test, the depressive-like behavior was confirmed by the increased immobility time in the TST. These results are in agreement with the clinical data showing a relationship among chronic pain and emotional disorders (Micó et al., 2006).

More interesting is the finding that SFN treatment inhibited the anxiety-like behavior associated with persistent neuropathic pain, as demonstrated by the increased number of entries and the percentage of time spent in open arms in the EPM test observed in SFN treated mice. In addition, the fact that the number of entries into closed arms was not affected by SFN excluded the possibility that its anxiolytic effects might be result from alterations of mice locomotor activity. Our results supported the demonstrated anxiolytic effects produced by SFN in several stress models (Muramatsu et al., 2013; Wu et al., 2016) and further revealed, for first time, the anxiolytic action of this compound in the anxiety-like behavior accompanying to chronic neuropathic pain. Moreover, the anxiolytic effects induced by SFN in Sham-operated mice evaluated in the EPM test confirmed its anxiolytic effects measured in the open field test (Wu et al., 2016).

Our data further revealed, for the first time, the anti-depressant effects induced by the repeated administration of SFN in animals with depressive-like behavior associated with neuropathic pain, as demonstrated by the decreased immobility time in the TST. These data are in agreement with the anti-depressant activities induced by some Nrf2 activators in several animal models of depression as well as to the depressive phenotype manifested in Nrf2 knockout mice (Wu et al., 2016; Yao et al., 2016; Zhang et al., 2017). In addition, the anti-depressant effects induced by SFN in our Sham-operated mice assessed in the TST confirmed the anti-depressant capacity of this treatment previously demonstrated in the forced swimming test (Wu et al., 2016). It’s important to mention that in contrast to the effects produced by several typical antidepressants, such as pregabalin that inhibited the anxiety-like behavior, but not the depressive-like response associated with neuropathic pain (La Porta et al., 2016), the repeated administration of SFN induced both anxiolytic and anti-depressant effects in animals with neuropathic pain. In brief, these results suggested the Nrf2 signaling pathway activation as a new therapeutic target for the treatment of anxiety and depression associated with chronic pain.

New theories of anxiety and depressive disorders revealed that beside inflammation, oxidative stress is also amply implicated in their development. Consequently, a significant decreased expression of the antioxidant enzymes activated by the Nrf2 signaling pathway in several anxiety- and depressive-like states has been demonstrated (Hashimoto, 2015; Khalifeh et al., 2015; Martín-Hernández et al., 2016). Thus, in order to evaluate the mechanisms implicated in the anxiolytic and anti-depressant properties of SFN in animals with chronic neuropathic pain, we evaluated its effects on the expression of Nrf2/HO-1/NQO1 signaling and CD11b/C in the prefrontal cortex and hippocampus of these animals. Our results showed a down-regulation of Nrf2 and HO-1 in prefrontal cortex and hippocampus, in addition to an up-regulation of CD11b/c in the hippocampus from animals with neuropathic pain, revealing that both brain areas are involved in the affective disorders associated to chronic pain. Interestingly, the fact that treatment with SFN normalized the diminished expression of Nrf2 and HO-1 and avoided the activation of microglia induced by nerve injury in hippocampus and prefrontal cortex might explain the anxiolytic and anti-depressant effects of SFN in animals with neuropathic pain. The lack of changes in the expression of CD11b/c in prefrontal cortex confirmed that sciatic nerve injury only activates microglia in specific brain areas (Riego et al., 2018).

Finally, our data also revealed that SFN improved the mechanical and thermal antiallodynic effects produced by morphine during neuropathic pain. Considering that neuropathic pain is difficult to treat with the most potent analgesic compounds, such as MOR agonists (Obara et al., 2009; Hervera et al., 2013), the fact that SFN potentiated the antiallodynic effects of morphine represented a very interesting stratagem for the treatment of sciatic nerve injury induced neuropathic pain. These effects might be linked with the normalization of the decreased expression of MOR induced by SFN in the spinal cord and/or hippocampus from sciatic nerve-injured mice. These data are consistent with the up-regulation of MOR induced by the activation of Nrf2 in animals with peripheral inflammation (Redondo et al., 2017) as well as with the neutralization of the down regulation of these receptors induced by CoPP in sciatic nerve-injured mice or diabetic animals (Hervera et al., 2013; Castany et al., 2016; Jurga et al., 2017). These results suggested that the modulation of the expression of MOR by SFN treatment is probably mediated via Nrf2/HO-1 activation. Nevertheless and considering that the pharmacological inhibition of microglia also increased the analgesic effects of MOR agonists during neuropathic pain (Zychowska et al., 2013), we cannot discard that the inhibition of microglial activation made by SFN in our animals might be also implicated in the increased antiallodynic effects of morphine in SFN pretreated animals.

## Conclusion

Our results revealed that treatment with SFN reduced allodynia and hyperalgesia induced by sciatic nerve injury and inhibited the anxiety and depressive-like behaviors associated with persistent neuropathic pain by normalizing oxidative stress, inhibiting microglial activation and MAPK phosphorylation in the specific areas evaluated. It is also interesting to note that treatment with SFN enhanced the antiallodynic activity of morphine by restoring the down regulation of MOR in the spinal cord and/or hippocampus from sciatic nerve-injured mice. In summary, this study showed that treatment with SFN might be an interesting approach for the management of persistent neuropathic pain and comorbidities associated, as well as to improve the analgesic actions of morphine in animals with neuropathic pain.

## Author Contributions

PF-C performed the behavioral tests. AR, GR, and SL performed the western blot assays. PF-C and OP performed the statistical analysis. PF-C and OP designed the study. OP wrote the manuscript. All authors contributed to manuscript revision, read and approved the submitted version.

## Conflict of Interest Statement

The authors declare that the research was conducted in the absence of any commercial or financial relationships that could be construed as a potential conflict of interest.
